# Comparison of 11C-choline Positron Emission Tomography/Computed Tomography (PET/CT) and Conventional Imaging for Detection of Recurrent Prostate Cancer

**DOI:** 10.7759/cureus.2966

**Published:** 2018-07-11

**Authors:** Yusuke Kawanaka, Kazuhiro Kitajima, Shingo Yamamoto, Yukako Nakanishi, Yusuke Yamada, Takahiko Hashimoto, Toru Suzuki, Shuken Go, Akihiro Kanematsu, Michio Nojima, Keitaro Sofue, Masakatsu Trsurusaki, Yukihisa Tamaki, Rika Yoshida, Koichiro Yamakado

**Affiliations:** 1 Radiology, Hyogo, Nishinomiya, JPN; 2 Radiology, Hyogo College of Medicine, Nishinomiya, JPN; 3 Urology, Hyogo College of Medicine, Nishinomiya, JPN; 4 Urology, Hyogo College of Medicine, Nisnomiya, JPN; 5 Radiology, Kobe University Graduate School of Medicine, Kobe, JPN; 6 Radiology, Kinki University Faculty of Medicine, Osakasayama, JPN; 7 Radiation Oncology, Shimane University Faculty of Medicine, Izumo, JPN; 8 Radiology, Shimane University, Faculty of Medicine, Izumo, JPN

**Keywords:** prostate cancer, recurrence, choline pet, ct, mri, bone scintigraphy

## Abstract

We aimed to compare ^11^C-choline positron emission tomography/computed tomography (PET/CT) with conventional imaging, including pelvic magnetic resonance imaging (MRI), contrast-enhanced chest, abdomen, and pelvic computed tomography (CT), and bone scintigraphy, for prostate cancer restaging. Thirty patients (median prostate-specific antigen [PSA: 11.8 ng/mL]) with suspected recurrent prostate cancer following definitive treatment underwent ^11^C-choline PET/CT and conventional imaging, including pelvic MRI, contrast-enhanced chest, abdomen, and pelvic CT, and bone scintigraphy. The results were compared with regard to patient- and lesion-based diagnostic performance for local recurrence, and for lymph node and bony metastases using receiver operating characteristic (ROC) analysis and McNemar’s test. Documented local recurrence and node and bony metastases were present in 11 (36.7%), 10 (33.3%), and 17 (56.7%) cases, respectively, of the enrolled patients. Patient-based sensitivity / specificity / accuracy / area under the ROC curve for ^11^C-choline-PET/CT for diagnosing local recurrence were 90.9% / 94.7% / 93.3% / 0.975 and for conventional imaging were 90.9% / 100% / 96.7% / 1.0. Those who underwent ^11^C-choline-PET/CT for node metastasis were 90.0% / 95.0% / 93.3% / 0.925 and for conventional imaging were 70.0% / 95.0% / 86.7% / 0.905. Those who underwent ^11^C-choline-PET/CT for bone metastasis were 94.1% / 92.3% / 93.3% / 0.991 and who underwent conventional imaging were 94.1% / 84.6% / 90.0% / 0.982. No significant differences were observed among them. The lesion-based detection rate of ^11^C-choline PET/CT for local recurrences and node and bone metastases as compared to conventional imaging was 92.9% (13/14) vs. 92.9% (13/14); 87.1% (27/31) vs. 54.8% (17/31); and 96.9% (219/226) vs. 90.3% (204/226) respectively, with significant differences noted for detection of node and bone lesions (p=0.0044 and p=0.00030, respectively). ^11^C-choline-PET/CT is more accurate in the detection of recurrent prostate cancer nodes and bony metastatic lesions compared to conventional imaging and has the advantage of restaging the disease in a single step.

## Introduction

In Western Europe and North America, prostate cancer is the most common tumor type in men and the second-most frequent cause of all deaths from cancer [1]. Although prostate cancer often develops slowly, it can later show an aggressive pattern and sometimes recurrence. For patients with recurrent prostate cancer, it is important to determine whether there is localized recurrent disease, metastasis to lymph nodes or bone, or a combination of localized recurrent and metastatic disease. This affects the subsequent management, such as consideration of salvage therapy for localized recurrence, systemic treatment for metastatic disease, or a combination of these. Computed tomography (CT), bone scintigraphy, pelvic magnetic resonance imaging (MRI), and trans-rectal ultrasound examinations have been traditionally used to determine the localization of recurrent or metastatic disease, though they lack the adequate sensitivity and accuracy. In recent years, positron emission tomography/computed tomography (PET/CT) using ^11^C- or ^18^F-choline has emerged as a promising molecular imaging tool and can provide full-body examination results in a single step [2-4].

No known study has been presented so far that directly compares the diagnostic capability of ^11^C-choline PET/CT with that of conventional imaging, including pelvic MRI, contrast-enhanced chest, abdomen, and pelvic CT, and bone scintigraphy, for restaging prostate cancer that has recurred following definitive treatment. Picchio et al. [5] compared ^11^C-choline PET with conventional imaging, including pelvic MRI, CT, bone scintigraphy, and trans-rectal ultrasound, for restaging of prostate cancer, but not PET/CT, though PET using ^11^C-choline was done while other modalities (pelvic MRI, CT, bone scintigraphy, trans-rectal ultrasound) were not performed in all of the enrolled patients. In addition, Beauregard et al. [6] compared ^11^C-choline PET/CT with conventional imaging, including pelvic CT and bone scintigraphy, for staging and restaging of prostate cancer, though pelvic MRI was not performed. Thus, the superiority of ^11^C-choline PET/CT over conventional imaging modalities, including pelvic MRI, contrast-enhanced chest, abdomen, and pelvic CT, and bone scintigraphy, for restaging of prostate cancer in patients who have undergone definitive treatment, has yet to be confirmed. The purpose of the present study was to clarify which method is preferable for the detection of local recurrence, as well as lymph node and bone metastasis in patients suspected to have recurrent prostate cancer.

## Materials and methods

This retrospective study was performed in accordance with the principles of the declaration of Helsinki. The institutional review board (Hyogo University Hospital, Japan) approved this retrospective study (No 2019). Informed consent was obtained from each patient after the procedure details were fully explained.

Patients

A total of 30 males (mean age 71.3 years, range 47-90 years) with suspected recurrent prostate cancer after receiving definitive treatment were included in this study and underwent ^11^C-choline PET/CT as well as conventional imaging, including pelvic MRI, contrast-enhanced chest, abdomen, and pelvic CT, and bone scintigraphy, at our institution between October 2015 and July 2017, with a maximum interval of two weeks between examinations. The median serum prostate-specific antigen (PSA) level in our cohort was 11.8 ng/mL (range 0.23-946 ng/mL). At the time of the examination, 26 (86.7%) of the 30 patients had received androgen-deprivation therapy. Additional patient details are shown in Table 1.

**Table 1:** Patient characteristics PSA: prostate specific antigen

Characteristics	Value
Age (years)	
Mean	71.3±9.0
Range	47～90
PSA (ng/mL)	
Mean	65.2±177.4
Range	0.23～946
Median	11.8
Previous treatment	
Radiotherapy	2
Surgery	1
Hormonal therapy	16
Radiotherapy＋hormonal therapy	10
Hormonal therapy＋chemotherapy	1

^11^C-choline PET/CT

^11^C-choline was synthesized using a commercial module, as described by Hara [7], and a CYPRIS-325R cyclotron (SHI, Tokyo, Japan). Acquisition of emission scan images from the mid-thigh to the head was started six minutes after intravenous injection of 3.0 MBq/kg body weight of ^11^C-choline. All PET/CT examinations were performed using a PET/CT scanner equipped with a 64-multidetector computed tomography device (Gemini TF64; Philips Medical Systems, Eindhoven, The Netherlands). The whole-body PET image acquisition in 3D mode was performed from the mid-thigh to the top of the head (1.5 minutes per bed position; 6-8 bed positions) and the obtained images were reconstructed using the ordered subsets expectation maximization reconstruction algorithm (33 subsets, three iterations, 4 mm per slice), with attenuation correction based on low-dose CT (120 kVp, 100 mA, slice thickness 2 mm, transverse field of view 600 mm), which was also used for anatomical correlations.

Conventional imaging

Pelvic MRI

The MRIs were performed using a Magnetom Avanto 1.5-T (Siemens Medical Solutions, Erlangen, Germany) or an Intera 1.5T (Philips Healthcare, Amsterdam, The Netherlands) system, equipped with a body coil for excitation and pelvic phased array coil for signal reception. Axial, coronal, and sagittal fast-spin-echo T2-weighted imaging (T2WI) were performed with a repetition time (TR)/echo time (TE) of 4000-4750 ms/110-120 ms, 3 mm slice thickness/0.3 mm gap, 28×22 cm field of view (FOV), and 228×256-256×320 matrix. T1-weighted imaging (T1WI) was performed in the axial plane with a spin-echo TR/TE of 500-550/9-10 ms, 3 mm slice thickness/0.3 mm gap, 28×22 cm FOV, and 228×256-256×320 matrix. Axial diffusion-weighted imaging (DWI) was performed along three orthogonal directions using spin-echo-type single-shot echo planar imaging with the following parameters: b value=0 and 1000 ms/mm^2^, TR/TE=3500-4500/70-75 ms, 3 mm slice thickness/0.3 mm gap, 42×32 cm FOV, and 128×108 matrix. In addition, coronal T1WI and T2WI were performed with a 3 mm slice thickness/0.3 mm gap, 59×30 cm FOV, and 228×256-256×320 matrix.

Contrast-enhanced CT

Pre-contrast and contrast-enhanced multi-detector CT (MDCT) images of the chest, abdomen, and pelvis were obtained using a 128-detector row CT device (SOMATOM Definition AS+, Siemens Medical Solutions, Erlangen, Germany) at 120 kV and effective mAs of 220 (CARE Dose4D), beam pitch of 0.6, collimation of 1.2 mm×32, and B31 + medium smooth + image reconstruction. Blood creatinine and the estimated glomerular filtration rate (eGFR) level were checked in all the patients before the examination. Iodinated contrast material (IopamironInj, Bayer Schering Pharma, Berlin, Germany) containing 300 mg of iodine per ml at a dose of 600 mg iodine per kilogram of body weight was intravenously administered using a power injector. The scan was started at 120 seconds after the injection to obtain venous phase images.

Bone scintigraphy

Whole-body bone scintigraphy was performed using a dual-head gamma camera (Forte, Hitachi, Tokyo, Japan) after intravenous administration of approximately 555 MBq of ^99m^Tc-methylene diphosphonate (^99m^MDP) (FujiFilm RI Pharma, Tokyo, Japan). Following the injection, the patient was orally hydrated and asked to void their bladder frequently as well as immediately prior to the scan. Total body images were obtained approximately three hours after administration of ^99m^MDP, with simultaneous anterior and posterior whole-body acquisition. Static additional acquisitions were acquired when needed. Single-photon emission computed tomography (SPECT) images were not acquired.

Imaging analysis

Two board-certified nuclear medicine physicians with no knowledge of the other imaging results or the final diagnosis interpreted the ^11^C-choline PET/CT findings, then used a five-point scale (1: definitely absent, 2: probably absent, 3: indeterminate, 4: probably present, 5: definitely present) to grade lesions in each patient based on consensus. Discordant readings by the two observers were resolved by a subsequent consensus review.

Focal ^11^C-choline activity in the prostate or a seminal vesicle greater than that in the adjacent background and not due to excreted radiotracer in urine was graded as 4 or 5 regardless of a corresponding structural abnormality. Lymph nodes were graded as 4 or 5 if distinct focal activity on the PET images was co-registered to a visible lymph node in CT images regardless of size. Focal skeletal sites of uptake above background marrow activity were graded as 4 or 5 unless inflammatory change, degenerative change, or fracture was evident. Semiquantitative analysis of abnormal radiotracer uptake for each lesion was also retrospectively performed using a maximum standardized uptake value (SUVmax), calculated as follows: SUV = volume of interest (VOI) radioactivity concentration (Bq/mL)/[injected dose (Bq)/patient weight (g)]. SUVmax, defined as the highest SUV value for pixels with the highest count within the VOI, was determined and recorded for the focal areas of uptake.

Two board-certified observers, a double board-certified nuclear medicine physician and radiologist, and a board-certified radiologist, with no knowledge of the other imaging results, or the final results, interpreted the conventional imaging findings and then used the same five-point scale to grade the lesions in each patient based on consensus. Discordant readings by the two observers for each modality were resolved by a subsequent consensus review.

The status of local recurrence was evaluated by pelvic MRI results. When T2WI findings were reviewed, a mass or soft-tissue area showing abnormal signal intensity was considered a positive finding (confidence score 4 or 5). In a review of DWI findings and apparent diffusion coefficient (ADC) maps, the presence of a lesion with high focal signal intensity on a DW image and low signal intensity on the ADC map relative to the background was considered to be a positive finding (score 4 or 5). Two positive interpretations of images in two sequences were given a confidence score of 5, while one positive interpretation in two sequences was given a score of 3 or 4. The number, location, and long-axis dimension of the suspected locally recurrent lesions were recorded.

The status of lymph node metastasis was evaluated by pelvic MRI as well as contrast-enhanced chest, abdomen, and pelvic CT findings. For scoring nodal status based on CT and MRI results, the following diagnostic criteria were used: short-axis diameter of node ≥10 mm, score 5; 8-9.9 mm, score 4; 5-7.9 mm, score 3; 1-4.9 mm, score 2; not seen, score 1. The number, location, and short-axis dimension of the suspected nodal metastatic lesions were recorded.

Bone metastasis status was also evaluated based on the combination of bone scintigraphy, contrast-enhanced chest, abdomen, and pelvic CT, and pelvic MRI findings. In bone scintigraphy images, a lesion was defined as malignant according to standard pathological criteria of intensity, localization, and the extension of ^99m^MDP uptake, with physiological uptake due to inflammatory change, degenerative change, or fracture excluded (score 4 or 5). In CT images, a lesion with osteoblastic or osteolytic change was graded as 4 or 5, unless inflammatory change, degenerative change, or fracture was evident. A lesion showing low signal intensity on T1WI and abnormal signal intensity on a DWI/ADC map is considered to be a positive finding (score 4 or 5). Inside the pelvis, three positive interpretations in three modalities were given a confidence score of five; two positive interpretations in three modalities a score of 4 or 5, and one positive interpretation a score of 3 or 4. Outside the pelvis, two positive interpretations of images in two sequences were given a confidence score of 5 and one positive interpretation in two sequences received a score of 3 or 4.

Gold standard

The final diagnosis was obtained based on histological confirmation, radiological imaging findings, or clinical follow-up results at least six months, including PSA level, MRI, CT, bone scintigraphy, and ^11^C-choline PET/CT findings.

Statistical analysis

To determine the utility of each imaging modality, receiver-operating-characteristic (ROC) analysis was employed. A ROC contrast estimation was utilized to compare the diagnostic capability of the two types of modalities on a per-patient basis. To test whether the area under the ROC curve (AUC) values were different, correlations of the testing methods were accounted for in the analysis. Tests for differences in sensitivity, specificity, and accuracy between the types of modalities were conducted using McNemar’s test. To calculate the sensitivity and specificity of each modality, scores of 4 and 5 were considered positive. A p-value of less than 0.05 was considered to indicate a statistically significant difference. All statistical analyses were performed using SAS software, version 9.3 (SAS Institute).

## Results

Local recurrence

**Figure 1:**
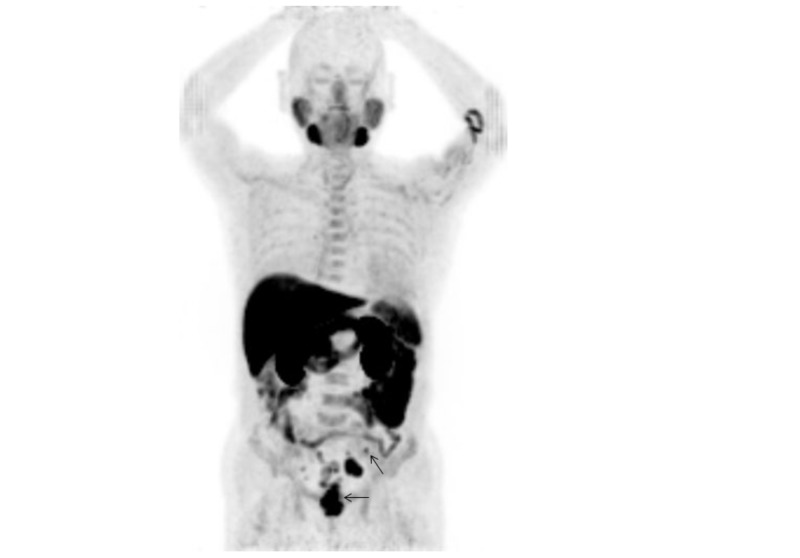
Maximum intensity projection of 11C-choline PET in a 79-year-old man with a PSA level of 24.8 ng/mL who underwent androgen-deprivation therapy for prostate cancer, in whom local recurrence in the prostate gland and pelvic node metastases were found (Case 1). Maximum intensity projection of 11C-choline positron emission tomography (PET) shows several abnormal 11C-choline uptake spots in the pelvis (arrows).

**Figure 2:**
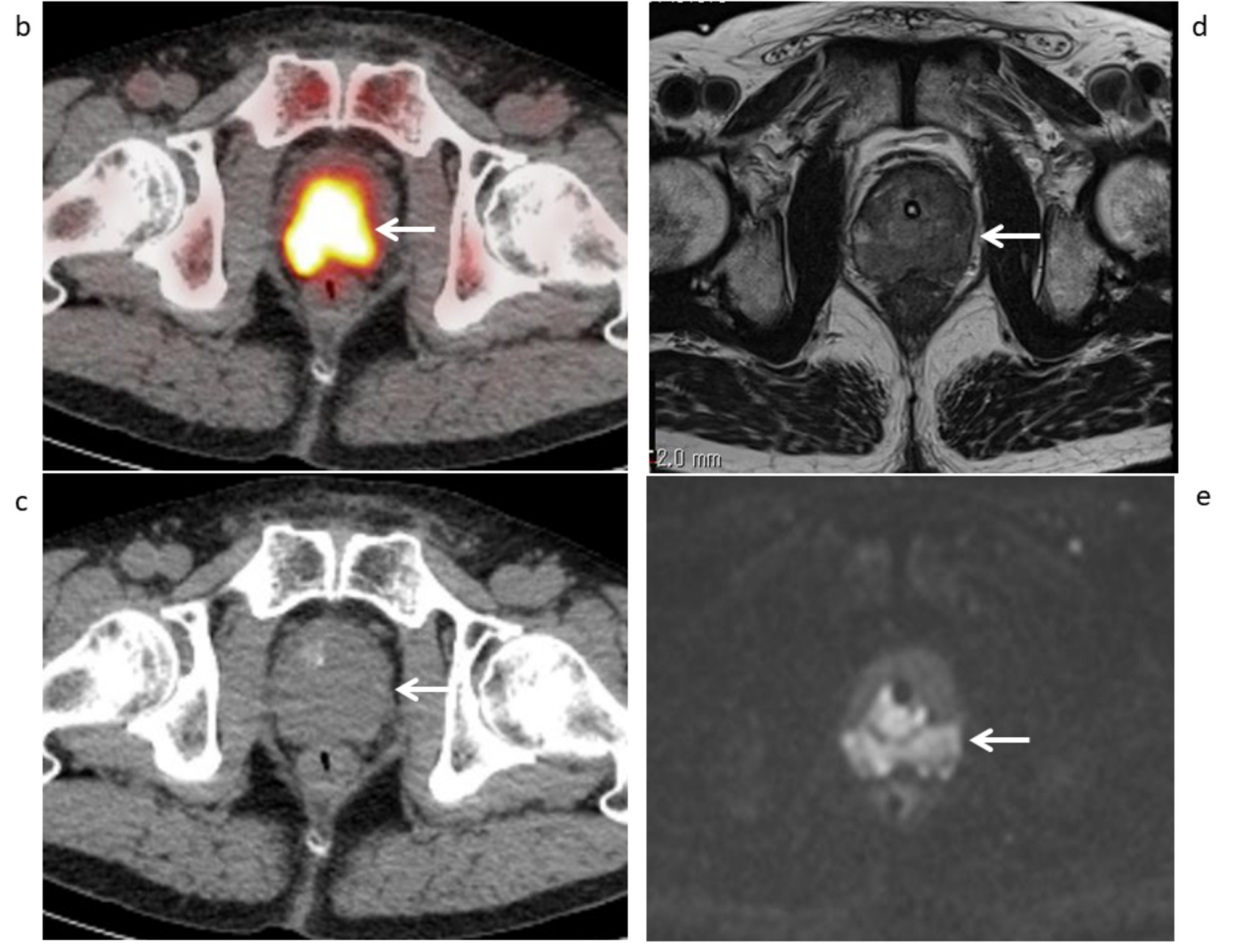
Axial 11C-choline PET/CT and pelvic MRI (Case 1) (b) Axial ^11^C-choline positron emission tomography/computed tomography (PET/CT) and (c) CT part show strong ^11^C-choline uptake (SUVmax 8.27) in the prostate gland (arrows), with a score of 5 for diagnosing local recurrence in the prostate gland. (d) Axial T2-weighted MR image shows hypointense area measuring 35×39 mm in the bilateral prostate gland with restricted diffusion on (e) DWI (arrows), with a score of 5 for diagnosing local recurrence in the prostate gland.

Eleven (36.7%) of the 30 patients were found to have a total of 14 local recurrent lesions (12 in the prostate gland, two in the seminal vesicles). The SUVmax values and long-axis diameters for these locally recurrent lesions as shown by ^11^C-choline PET/CT and conventional imaging were 6.74±2.92 (1.65-12.91) and 19.9±8.6 mm (9-35 mm), respectively. The patient- and lesion-based sensitivities of ^11^C-choline PET/CT as compared to conventional imaging were 90.9% (10/11) vs. 90.9% (10/11), and 92.9% (13/14) vs. 92.9% (13/14), respectively (Table 2-3). Case 1 shows that local recurrence in the prostate gland could be diagnosed by both  ^11^C-choline PET/CT (Figures 1-2) and pelvic MRI (Figure 2).

**Table 2:** Patient-based diagnostic result of 11C-choline PET/CT and conventional imaging AUC: area under the receiver-operating-characteristic curves; CI: confidence interval; PET/CT: positron emission tomography/computed tomography

	Sensitivity	Specificity	Accuracy	AUC
	95%CI	95%CI	95%CI	95%CI
Local recurrence				
^11^C-choline PET/CT	90.9%	94.7%	93.3%	0.975
	73.1～100%	84.4～100%	84.4～100%	0.85-0.996
Conventional imaging	90.9%	100%	96.7%	1.0
	73.1～100%	100%	90.2～100%	1.0
p value	1.0	1.0	1.0	0.30
Lymph node metastasis				
^11^C-Choline PET/CT	90.0%	95.0%	93.3%	0.925
	41.6～98.4 %	85.4～100%	84.4～100%	0.596-0.990
Conventional imaging	70.0%	95.0%	86.7%	0.905
	53.6～86.4%	85.4～100%	73.8～98.8%	0.615-0.983
p-value	0.48	1.0	0.48	0.28
Bone metastasis				
^11^C-Choline PET/CT	94.1%	92.3%	93.3%	0.991
	82.9～100%	77.8～100%	84.4～100%	0.918-0.999
Conventional imaging	94.1%	84.6%	90.0%	0.982
	82.9～100%	65.0～100%	79.3～100%	0.902-0.997
p-value	1.0	1.0	1.0	0.39
All lesions				
^11^C-Choline PET/CT	92.0%	100%	93.3%	0.964
	81.4～100%	100%	84.4～100%	0.828-0.993
Conventional imaging	92.0%	80.0%	90.0%	0.976
	81.4～100%	44.9～100%	79.3～100%	0.812-0.997
p value	1.0	1.0	1.0	0.71

One false-negative case in the ^11^C-choline PET/CT findings was a tiny (9 mm) local recurrent lesion in the prostate gland that showed faint ^11^C-choline uptake (SUVmax 1.65). Another false-negative case which was determined by conventional imaging was a local recurrence in the seminal vesicles measuring 14 mm, which showed moderate ^11^C-choline uptake (SUVmax 4.53). One false-positive case was choline nonspecific uptake (SUVmax 4.84) in the prostate gland shown by ^11^C-choline PET/CT (Figures 1-2).

Lymph node metastasis

**Figure 3:**
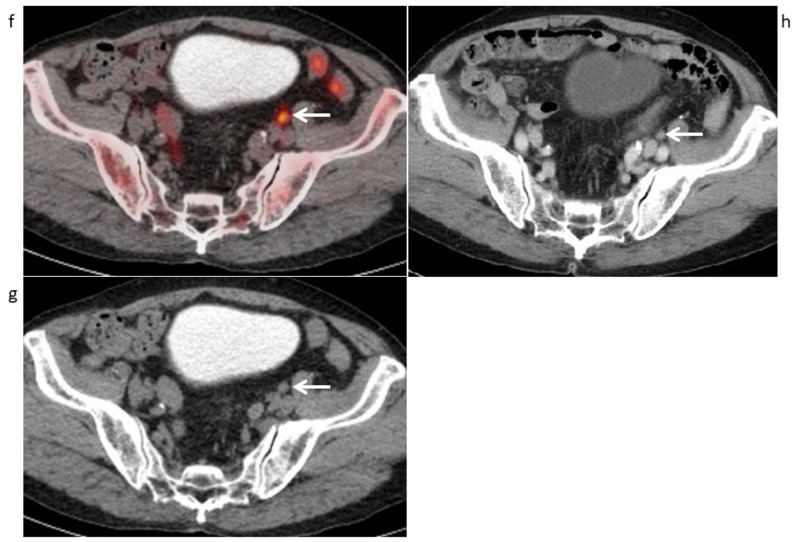
Axial 11C-choline PET/CT and contrast-enhanced CT (Case 1) (f) Axial 11C-choline PET/CT and (g) CT part show mild swelling measuring 7 mm in short diameter and abnormal 11C-choline uptake (SUVmax 3.42) of the left external iliac node (arrow), with a score of 5 for diagnosing lymph node metastasis. (h) Contrast-enhanced CT shows mild swelling of the left external iliac node measuring 7 mm in short diameter (arrow), with a score of 3 for diagnosing lymph node metastasis.

Ten (33.3%) of the 30 patients had a total of 31 metastatic lymph nodes (six external iliac, five internal iliac, two obturator, four common iliac, eight abdominal para-aortic, three mediastinal, and three supraclavicular). The SUVmax values and short-axis diameters of those metastatic nodes as shown by ^11^C-choline PET/CT and conventional imaging were 5.48±3.21 (0.89-13.9) and 10.1±5.8 mm (4-25 mm), respectively. The patient- and lesion-based sensitivity of 11C-choline PET/CT as compared to conventional imaging was 90.0% (9/10) vs. 70.0% (7/10), and 87.1% (27/31) vs. 54.8% (17/31), respectively (p=0.48 and p=0.0044, respectively) (Tables 2-3). Case 1 shows that a tiny pelvic lymph node metastasis could be diagnosed by ^11^C-choline PET/CT, but not contrast-enhanced CT (Figure 3).

Four false-negative cases were determined by ^11^C-choline PET/CT and consisted of two tiny para-aortic metastatic nodes measuring 4 and 5 mm, respectively (one tiny internal iliac node measuring 6 mm and one tiny external iliac node measuring 6 mm). Those four nodal lesions showed no or faint ^11^C-choline uptake (SUVmax: 0.89, 1.21, 1.49, and 1.77). Fourteen false-negative cases were determined by conventional imaging and were all tiny lesions whose short-axis diameter measured less than 8 mm. One false-positive case determined by ^11^C-choline PET/CT showed ^11^C-choline nonspecific uptake (SUV max: 3.61) in the inguinal node. One false-positive case was also determined by conventional imaging and had non-specific enlarged hilar and mediastinal nodes measuring larger than 10 mm.

**Table 3:** Lesion-based diagnostic sensitivity of 11C-choline PET/CT and conventional imaging PET/CT: Positron emission tomography/computed tomography, CI: confidence interval, SUVmax: maximum standard uptake value

	^11^C-choline PET/CT	Conventional imaging	p value
Local Recurrence			
Sensitivity	92.9%	92.9%	1
95% CI	79.4～100%	79.4～100%	
SUVmax of all 14 lesions	6.74±2.92		
Lesion long diameter of all 14 lesions		19.9±8.6 mm	
Lymph node metastasis			
Sensitivity	87.1%	54.8%	0.0044
95% CI	75.3～98.9%	37.3～72.4%	
SUVmax of all 31 lesions	5.48±3.21		
Lesion short diameter of all 31 lesions		10.1±5.8 mm	
Bone metastasis			
Sensitivity	96.9%	90.3%	0.00030
95% CI	94.6～99.2%	86.4～94.1%	
SUVmax of all 226 lesions	5.23±2.59		
All lesions			
Sensitivity	95.6%	86.3%	<0.0001
95% CI	93.1～98.0%	82.3～90.4%	

Bone metastasis

**Figure 4:**
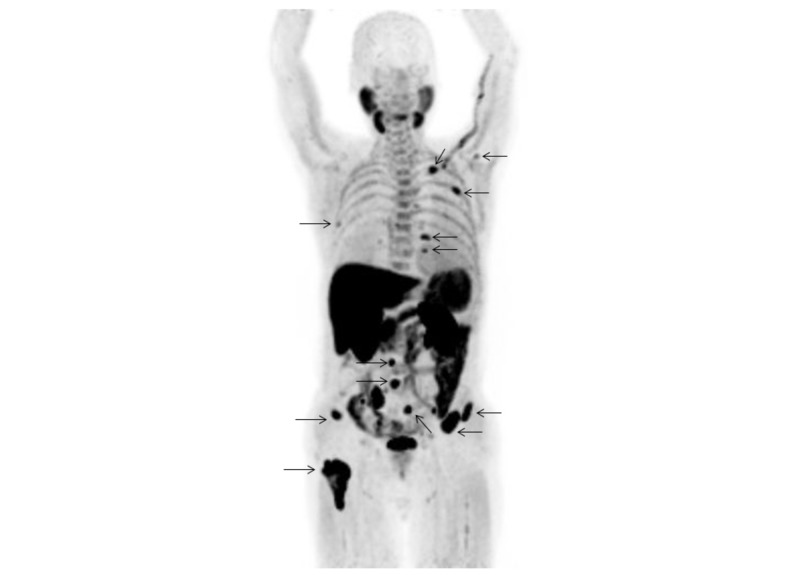
Maximum intensity projection of 11C-choline PET in a 70-year-old man with a PSA level of 10.13 ng/mL who underwent radiotherapy and androgen-deprivation therapy for prostate cancer, in whom multiple bone metastases were found (Case 2) (a) Maximum intensity projection of ^11^C-choline PET shows many abnormal ^11^C-choline uptake spots in the whole-body bones with the ribs, clavicle, lumbar spine, sacrum, bilateral iliac bones, and right lower extremity (arows).

**Figure 5:**
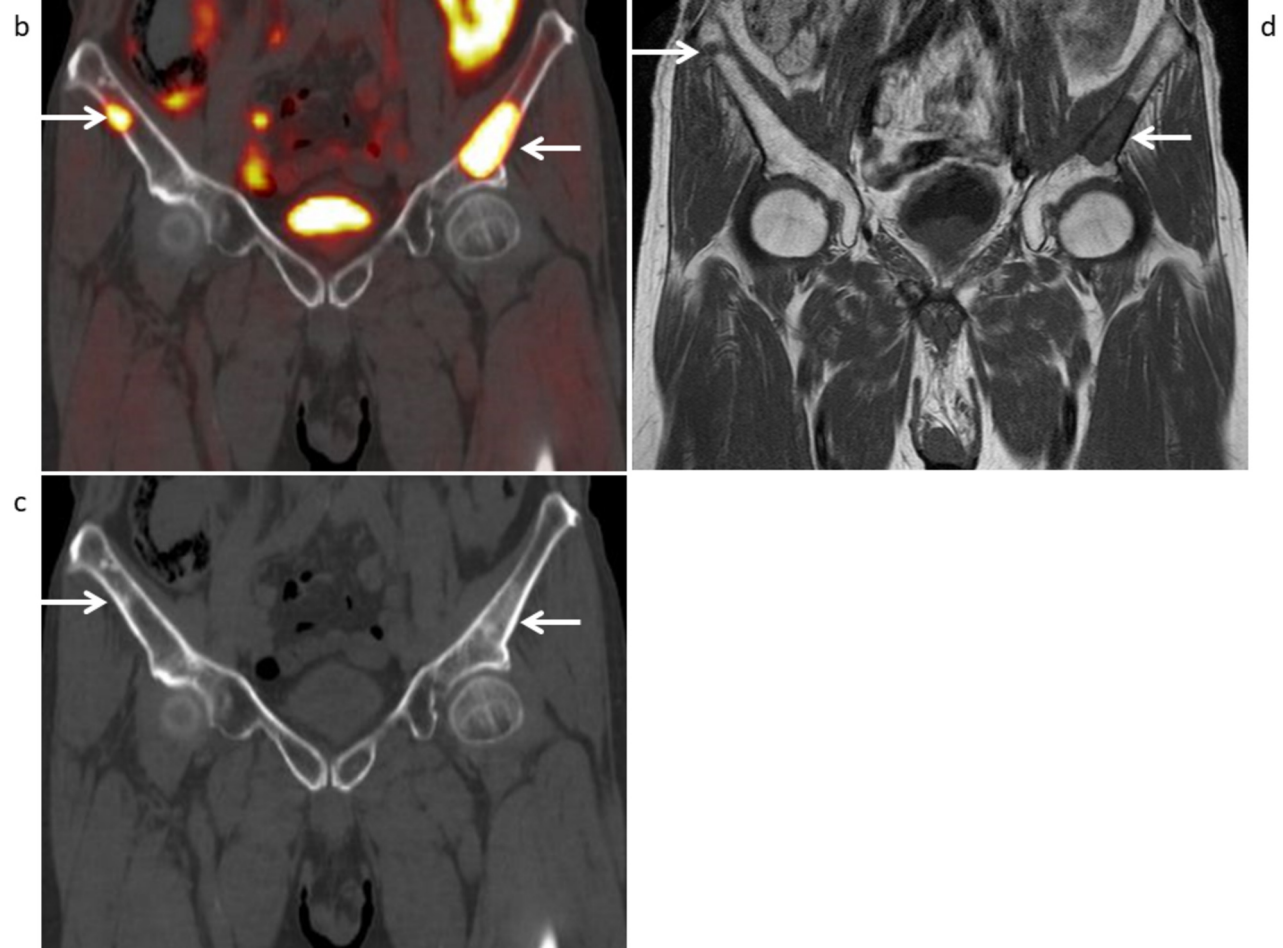
Coronal 11C-choline PET/CT and pelvic MRI (Case 2) (b) Coronal ^11^C-choline PET/CT and (c) CT part (bone condition) show strong ^11^C-choline uptake (SUVmax 11.32 (left) and 5.91 (right)) of the osteoblastic change in the bilateral iliac bones (arrows), with a score of 5 for diagnosing bone metastasis. (d) Coronal T1-weighted MR image shows hypointense areas in the bilateral iliac bones (arrows), with a score of 5 for diagnosing bone metastasis.

**Figure 6:**
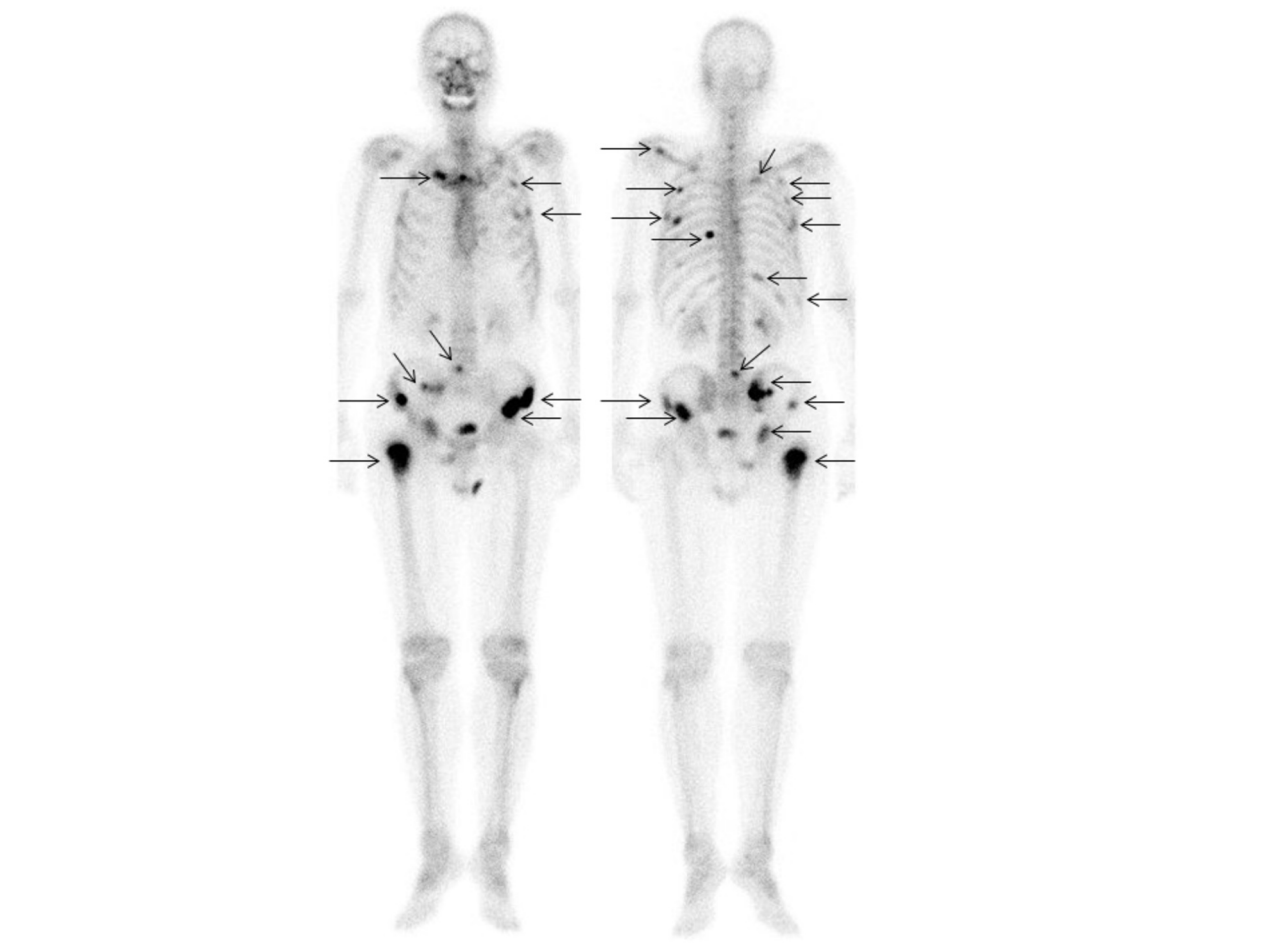
99mMDP bone scintigraphy (Case 2) Anterior and posterior whole-body views of bone scintigraphy shows strong 99mMDP uptake in the ribs, clavicle, lumbar spine, sacrum, bilateral iliac bones, and right lower extremity (arrows), with a score of 5 for diagnosing bone metastasis.

Seventeen (56.7%) of the 30 patients had a total of 226 metastatic bone lesions (16 cervical spine, 36 thoracic spine, 21 lumbar spine, 17 sacrum, 22 iliac bone, 16 pubic, 16 ischium, 15 acetabulum, six sternum, four clavicle, 22 ribs, 13 scapula, eight upper extremities, 11 lower extremities, and three skull). The mean SUVmax value of the 226 bone metastases shown by ^11^C-choline PET/CT was 5.23±2.59 (range 1.77-14.62). The patient- and lesion-based sensitivity of ^11^C-choline PET/CT as compared to conventional imaging was 94.1% (16/17) vs. 94.1% (16/17), and 96.9% (219/226) vs. 90.3% (204/226), respectively (p=1.0 and p=0.00030, respectively) (Table 2-3). Inside the pelvis, the lesion-based sensitivity of ^11^C-choline PET/CT and conventional imaging for the sacrum, iliac bone, pubic, ischium, and acetabulum was the same (84/86, 97.7%). On the other hand, outside the pelvis, lesion-based sensitivity of ^11^C-choline PET/CT was 96.4% (135/140) as compared to 85.7% (120/140) for conventional imaging (p=0.00030) of the cervical spine, thoracic spine, lumbar spine, sternum, clavicle, ribs, scapula, upper extremities, lower extremities, and skull. Case 2 shows that multiple bone metastases could be detected by ^11^C-choline PET/CT (Figures 4-5), pelvic MRI (Figure 5), and bone scintigraphy (Figure 6). Table 4 presents the distribution and visualization rates of bone metastases shown by ^11^C-choline PET/CT and conventional imaging.

**Table 4:** Distribution and visualization rates of bone metastasis between 11C-choline PET/CT and conventional imaging PET/CT: Positron emission tomography/computed tomography

Location	Lesion no.	Sensitivity of ^11^C-choline PET/CT	Sensitivity of Conventional imaging	p value
Cervical spine	16	93.8%	93.8%	1
Thoracic spine	36	94.4%	94.4%	1
Lumbar spine	21	90.5%	90.5%	1
Sacrum	17	94.1%	94.1%	1
Iliac bone	22	100%	95.5%	1
Pubic	16	100%	100%	1
Ischium	16	93.8%	93.8%	1
Acetabulum	15	100%	100%	1
Sternum	6	100%	83.3%	1
Clavicle	4	100%	75.0%	1
Ribs	22	100%	77.3%	0.074
Scapula	13	100%	76.9%	0.25
Upper extremities	8	100%	100%	0.48
Lower extremities	11	100%	81.8%	0.48
Skull	3	100%	66.7%	1
Total	226	96.9%	90.3%	0.00030

**Table 4:** Distribution and visualization rates of bone metastasis between 11C-choline PET/CT and conventional imaging PET/CT: Positron emission tomography/computed tomography

Location	Lesion no.	Sensitivity of ^11^C-choline PET/CT	Sensitivity of Conventional imaging	p value
Cervical spine	16	93.8%	93.8%	1
Thoracic spine	36	94.4%	94.4%	1
Lumbar spine	21	90.5%	90.5%	1
Sacrum	17	94.1%	94.1%	1
Iliac bone	22	100%	95.5%	1
Pubic	16	100%	100%	1
Ischium	16	93.8%	93.8%	1
Acetabulum	15	100%	100%	1
Sternum	6	100%	83.3%	1
Clavicle	4	100%	75.0%	1
Ribs	22	100%	77.3%	0.074
Scapula	13	100%	76.9%	0.25
Upper extremities	8	100%	100%	0.48
Lower extremities	11	100%	81.8%	0.48
Skull	3	100%	66.7%	1
Total	226	96.9%	90.3%	0.00030

Eight false-negative cases shown by ^11^C-choline PET/CT were composed of one cervical spine, two thoracic spine, two lumbar spine, one sacrum, and one ischium case, while 22 false-negative cases shown by conventional imaging were composed of one cervical spine, two thoracic spine, one lumbar spine, one sacrum, one iliac bone, one ischium, one sternum, one clavicle, five ribs, three scapula, two upper extremities, two lower extremities, and one skull case. One false-positive case shown by ^11^C-choline PET/CT had moderate ^11^C-choline uptake (SUVmax: 4.13) observed in a normal thoracic vertebra, and false-positive cases shown by conventional imaging were degenerative changes in the cervical and lumbar vertebra, which showed strong ^99m^MDP uptake and osteoblastic changes in CT images.

All lesions

There was a total of 271 recurrent or metastatic lesions in 25 (83.3%) of the 30 patients. Patient-based analysis showed that the sensitivity, specificity, accuracy, and AUC of ^11^C-choline PET/CT were 92.0% (23/25), 100% (5/5), 93.3% (28/30), and 0.964, respectively, whereas those of conventional imaging were 92.0% (23/25), 80.0% (4/5), 90.0% (27/30), and 0.976, respectively (Table 2). No significant differences were observed among those four parameters. The lesion-based sensitivity of ^11^C-choline PET/CT as compared to conventional imaging was 95.6% (259/271) vs. 86.3% (234/271) (p<0.0001) (Table 3).

## Discussion

The present is the first study to directly compare the diagnostic capability of ^11^C-choline PET/CT with that of conventional imaging, including pelvic MRI, contrast-enhanced chest, abdomen, and pelvic CT, and bone scintigraphy, for restaging prostate cancer in patients who have undergone definitive treatment. We found that the lesion-based sensitivity of ^11^C-choline PET/CT for detection of node and bone metastases in patients with recurrent prostate cancer was significantly higher as compared to conventional imaging modalities, with ^11^C-choline PET/CT and conventional imaging exhibiting equal sensitivity for the diagnosis of local recurrent disease. Although patient-based analysis did not reveal a significant difference between the two types of imaging, our results confirm those of a previous study, in which Picchio et al. [5] demonstrated that the patient-based sensitivity of ^11^C-choline PET and conventional imaging for restaging prostate cancer was 47% and 49%, respectively.

In Western countries, ^11^C- and ^18^F-choline PET/CT have been successfully used for restaging prostate cancer in patients with biochemical disease recurrence after undergoing deﬁnitive therapy, especially in those with a PSA level >1.0 ng/mL [2, 4, 8]. Another systematic review and meta-analysis of 19 selected studies with a total of 1,555 patients reported a pooled sensitivity of 85.6% and a pooled specificity of 92.6% [9]. Picchio et al. concluded that the routine use of ^11^C-choline PET/CT for restaging prostate cancer after a radical prostatectomy cannot be recommended for patients with a PSA value <1 ng/mL [8]. Furthermore, they noted that in addition to the PSA value, PSA doubling time and other clinical and pathological features, such as a locally advanced tumor (pT3a-T4) or nodal involvement at the initial staging, should be considered when considering patients for ^11^C-choline PET/CT.

The current gold standard for the diagnosis of a locally recurrent prostate cancer in patients who have undergone a radical prostatectomy procedure and radiation therapy is a dynamic gadolinium contrast-enhanced MRI [10-11]. Dynamic contrast-enhanced MRI was not performed in the present cases, thus the sensitivity of conventional imaging for local recurrence may have been underestimated. Kitajima et al. [3] compared multi-parametric MRI modalities, including T2WI, DWI, and dynamic contrast-enhanced imaging, with ^11^C-choline PET/CT for detection of local recurrence after a radical prostatectomy in 87 patients and reported that patient-based sensitivity, specificity, and accuracy of multi-parametric MRI were 88.5%, 84.6%, and 87.4%, respectively, whereas they were 54.1%, 92.3%, and 65.5%, respectively, for ^11^C-choline PET/CT.

Evaluation of lymph node metastasis is crucial for restaging patients with PSA failure after treatment. In a study of 25 prostatectomy patients with PSA failure using pelvic and retroperitoneal lymphadenectomy histopathology results as a reference [12], the values for sensitivity, speciﬁcity, positive predictive value, negative predictive value, and accuracy of ^11^C-choline PET/CT for depicting nodal metastasis were 100%, 66%, 90%, 100%, and 92% respectively, in patient-based analysis and 64%, 90%, 86%, 72%, and 77% respectively, in lesion-based analysis. The low negative predictive value for lesion-based analysis in that the study indicated the limited performance of ^11^C-choline PET/CT in detecting microscopic nodal metastasis, whereas the high positive predictive value is an important result for facilitation of appropriate treatment. Kitajima et al. [3] also compared pelvic MRI and ^11^C-choline PET/CT results for detecting pelvic nodal metastasis following a radical prostatectomy in 70 patients, and they reported that the patient-based sensitivity, specificity, and accuracy values for pelvic MRI were 64.0%, 85.0%, and 70.0%, respectively, whereas those of ^11^C-choline PET/CT were 90.0%, 100%, and 92.9%, respectively. In the present study, we also noted the superiority of ^11^C-choline PET/CT as compared to MRI results.

In the present patient series, the ability to diagnose metastasis to the bone outside of the pelvis was significantly different between ^11^C-choline PET/CT and conventional imaging (bone scintigraphy and CT), whereas with regard to metastasis to bone inside the pelvis, it was the same between the imaging types because of the features of MRI. Bone scintigraphy is a low-cost whole-body clinical examination method commonly used to detect skeletal involvement in patients with prostate cancer, though it is known to be inferior to ^11^C-choline PET/CT for the detection of bone metastases. A previous meta-analysis demonstrated that the pooled sensitivity and specificity of ^11^C-choline PET/CT were 0.91 (95% confidence interval (CI): 0.83–0.96) and 0.99 (95% CI: 0.93–1.00), respectively, while those values for bone scintigraphy were 0.79 (95% CI: 0.73–0.83) and 0.82 (95% CI: 0.78–0.85), respectively [13]. Similar to the findings of the present cohort, Kitajima et al. [3] reported equally excellent accuracy for the diagnosis of pelvic bone metastases after undergoing a radical prostatectomy in 95 patients, with values for patient-based sensitivity, specificity, and accuracy for pelvic MRI found to be 87.5%, 96.2%, and 94.7%, respectively, whereas those for ^11^C-choline PET/CT were 81.3%, 98.7%, and 95.8%, respectively. Although the superiority of ^11^C-choline PET/CT as compared to the whole-body MRI is controversial [13-14] and further analysis is needed, we consider that it is necessary to use ^11^C-choline PET/CT or a whole-body MRI for a careful examination to check for whole-body bone metastasis. In contrasting reports, a group reported the superiority of the whole-body MRI [13], while another found that ^11^C-choline PET/CT was superior [14].

Our study has some limitations, including the small number of patients enrolled from a single institution. A prospective multicenter trial with a larger cohort would help to clarify the exact role of ^11^C-choline PET/CT in clinical decision-making and long-term outcomes in clinical settings. Also, the enrolled population was heterogeneous, as it included treatment-naive patients, as well as those who did or did not undergo hormonal treatment. Such heterogeneity likely introduced confounding factors into the analysis. The follow-up period was relatively short. In addition, the ideal gold standard for any analysis would be a histological confirmation of obtained findings. On the other hand, clinical follow-up results are considered to be valid for evaluations of diagnostic accuracy and response to therapy, and it would have been unethical to investigate all PET/CT-detected lesions with the use of an invasive procedure. Although the positive findings are easy to confirm, negative findings only indicate that it was not possible to acquire positive findings during a follow-up examination, making it uncertain whether the findings are truly negative. Furthermore, new and more sensitive PET tracers for prostate cancer, such as ^18^F-FACBC and ^68^Ga-PSMA, have been recently introduced for clinical use in Western countries [4], though they are not yet available in Japan. One study demonstrated that ^68^Ga-PSMA PET/CT showed a higher detection rate than ^11^C-choline PET for lymph nodes as well as bone lesions in patients with prostate cancer [15]. Finally, SPECT/CT findings were not available in the present study. A hybrid imaging method (SPECT/CT) may increase the sensitivity and specificity of bone scintigraphy by identifying benign bone conditions with increases in bone turnover and whenever equivocal findings of planar bone imaging are noted [16].

## Conclusions

We found that the lesion-based sensitivity of ^11^C-choline PET/CT for detection of node and bone metastases in patients with recurrent prostate cancer was significantly higher as compared to the conventional imaging modalities, with results showing equal sensitivity for the diagnosis of local recurrent disease between ^11^C-choline PET/CT and conventional imaging. ^11^C-choline-PET/CT is more useful for detecting node and bony metastatic lesions from recurrent prostate cancer than conventional imaging, and has an additional advantage in restaging disease in a single step.
